# A Broad-Spectrum Sweet Taste Sensor Based on Ni(OH)_2_/Ni Electrode

**DOI:** 10.3390/s18092758

**Published:** 2018-08-22

**Authors:** Yuezhong Mao, Shiyi Tian, Shuanglin Gong, Yumei Qin, Jianzhong Han, Shaoping Deng

**Affiliations:** School of Food Science and Biotechnology, Zhejiang Gongshang University, Zhejiang 310018, China; myz-001@163.com (Y.M.); daringame@163.com (S.G.); yqin@zjsu.edu.cn (Y.Q.); hanjz99@zjsu.edu.cn (J.H.); spdeng@zjgsu.edu.cn (S.D.)

**Keywords:** taste sensor, Ni(OH)_2_/Ni, characteristic value, sweet substances, sweetness

## Abstract

A broad-spectrum sweet taste sensor based on Ni(OH)_2_/Ni electrode was fabricated by the cyclic voltammetry technique. This sensor can be directly used to detect natural sweet substances in 0.1 M NaOH solution by chronoamperometry method. The current value measured by the sensor shows a linear relationship with the concentration of glucose, sucrose, fructose, maltose, lactose, xylitol, sorbitol, and erythritol (R^2^ = 0.998, 0.983, 0.999, 0.989, 0.985, 0.990, 0.991, and 0.985, respectively). Moreover, the characteristic value of this sensor is well correlated with the concentration and relative sweetness of eight sweet substances. The good correlation between the characteristic value of six fruit samples measured by the sensor and human sensory sweetness measured by sensory evaluation (correlation coefficient = 0.95) indicates that it can reflect the sweetness of fruits containing several sweet substances. In addition, the sensor also exhibits good long-term stability over 40 days (signal ratio fluctuation ranges from 91.5% to 116.2%). Thus, this broad-spectrum sensor is promising for sweet taste sensory application.

## 1. Introduction

Human taste sensations can be divided into five primary qualities: bitter, sour, salty, sweet, and umami [1]. Sweet is considered to be the most essential basic taste and has the high relation with the human body [2,3]. Sweetness is a favorite natural taste for babies [4]. Infants preferred sweet taste over water at the age of 3, 6, and 12 months [5]. In adults, there is a small but significant association between the subjects’ frequency of consumption of sweet foods [6]. In the food industry, sweet substances and sweet taste are also very important, especially in the beverage and fruit juice area. The appropriate sweet substance concentration can contribute to the appealing taste of products. 

Currently, most popular methods of sweet taste detection are based on human sensory evaluation. The human sensory evaluation has been widely used over the world to evaluate sweet taste sensation and other taste sensations of different food, such as fruit, juice, beverage, and other liquid food [7,8,9,10]. Nevertheless, human sensory evaluation also has some flaws, such as being time-consuming and expensive, and might vary depending on tester’s daily conditions. Thus, some appropriate novel methods are expected to detect the sweet taste.

In recent years, many sensors have been developed for the detection of single sweet substance. Xiaohui Zhang et al. [11] fabricated a nonenzymatic electrochemical glucose biosensor based on the Ni/NiO-rGO. This sensor could be utilized for quantification of glucose from 29.9 μM to 6.44 mM. Lucian Rotariu et al. [12] fabricated a microbial biosensor based on immobilised microorganisms. This biosensor was used for the selective monitoring of sucrose in the presence of glucose, using a second anti-interference enzymatic layer with glucose oxidase (GOD) and catalase (CAT). Soomro et al. [13] fabricated an enzyme free electrochemical glucose sensor based on the direct modification of glassy carbon electrode (GCE) with Au NCs prepared using a simple galvanic replacement reaction. Deepti Sharma et al. [14] fabricated a novel electrochemical glucose sensor employing an interdigitated array (IDA) of 1:1 aspect ratio carbon nanoelectrodes for the electrochemical-enzymatic redox cycling of redox species (ferricyanide/ferrocyanide) between glucose oxidase (GOx) and the two comb-shaped nanoelectrodes of the IDA. Jiewu Cui et al. [15] developed a glucose sensor by integration of glucose oxidase (GOx) with a gold nanowires array (AuNWA) by cross-linking with a mixture of glutaraldehyde (GLA) and bovine serum albumin (BSA). Yagmur Koskun et al. [16] synthesized a novel glucose sensor, highly sensitiveactivated carbon (AC) decorated monodisperse nickel, and palladium alloy nanocomposites modified glassy carbon electrode. Marx et al. [17] developed a fructose bioelectrode based on nanofibers of electrospun gold with immobilized fructose dehydrogenase. Nguyen et al. [18] developed a lactose biosensor by co-immobilizing b-galactosidase and glucose oxidase on microelectrodes pre-modified with Pt/graphene/P (1,5-DAN). Odaci et al. [19] designed a new bi-enzymatic system by co-immobilization of α-glucosidase and pyranose oxidase for maltose analysis. Liang Feng et al. [20] fabricated a biosensor based on molecularly imprinted electrosynthesized polymers for determination of sorbitol. Gualandi et al. [21] developed a device for the amperometric detection of sugars in flow systems based on the electrochemical deposition of a Co/Al layered double hydroxide on a Pt electrode. These sensors show a good relationship and high selectivity to the sweet substances, and have been used for detection of the concentration of one special substance in blood, serum, and interstitial fluid for the purpose of biochemical detection. However, for the sweet taste sensor, broad-spectrum responses and responses to several sweet substances at the same time are more important and necessary.

On the other hand, some sensors have been designed for sweet taste detection and were named as the sweet taste sensor. Habara et al. [22] developed novel lipid/polymer membranes, such as phosphoric acid di-*n*-hexadecyl ester and tetradodecylammoniumbromid, and a plasticizer, dioctyl phenylphosphonate. This new membrane can detect the sucrose and other sugar alcohol. Yasuura et al. [23] developed a novel lipid/polymer membrane sensor, which was composed of TDAB, PVC and PTEH for saccharin sodium and acesulfame potassium detection. These sweet taste sensors have many advantages. They can detect two or more kinds of sweet substances and show nearly zero responses to other basic taste samples. The sensors have high selectivity and concentration-dependent responses to several sweet substances. These sweet taste sensors also have some disadvantages, such as the fact that the correlation with human sensory for glucose and sorbitol is not very good, the sensor’s working life is not very long (about 3000 times), and it cannot be used in some special food samples (alcohol-soluble and eater-soluble drink). 

Previous studies showed that Ni(OH)_2_ and related sensor can detect organic compounds in the alkaline [24,25,26,27,28]. In these previous studies, the Ni(OH)_2_ and related sensor was used to detect the glucose. In our recent research work, we found that the Ni(OH)_2_ sensor can also respond to some other sugars under certain experimental conditions, such as sucrose, maltose, and fructose. We propose that this characteristic can be applied to sweet taste sensor. Thus, in this paper, the Ni(OH)_2_/Ni sensor was fabricated by cyclic voltammetry technique and was used to detect eight kinds of natural sweet substances. The properties of the sweet taste sensor and detection of different natural sweet substances were discussed. The characteristic value of this sensor and the correlation with sweet substances’ relative sweetness were studied. Moreover, the application of natural fruits’ sweetness detection and correlation with human sensory sweetness were researched. In addition, the stability of the sensor was also described in this study. 

## 2. Materials and Methods

### 2.1. Reagents and Materials

Nickel disk electrode (diameter of 2 mm and length of 20 mm) was acquired from Tianjin Aida Hengsheng Technology Co., Ltd. (Tianjin, China). Potassium ferricyanide, potassium chloride, sodium hydroxide, glucose, sucrose, fructose, lactose, maltose, xylitol, sorbitol, and erythritol were purchased from Sinopharm Chemical Reagent Co., Ltd. (Shanghai, China). All the chemical reagents were used as received. All solutions were prepared using Milli-Q water, with resistivity of 18.2 MΩ·cm using a Millipore system. 

### 2.2. Fabrication and Electrochemical Measurements of Sweet Taste Sensor 

The nickel disk electrode (diameter of 2 mm) was polished to mirror finish by Al_2_O_3_ powder (diameter of 1 μm and 50 nm), and ultrasonically cleaned in ethanol and deionized water successively. Then, nickel disk electrode was immersed into 6 M NaOH solution and the potential cycling was taken between 0 and 500 mV at a scan rate of 100 mV/s for 500 cycles [29]. 

The electrochemical measurements were performed on a CHI 1030 workstation (CH Instruments, Shanghai, China) with conventional three electrodes system. Nickel disk electrode (diameter of 2 mm) was used as the working electrode. Platinum disk electrode (diameter of 5 mm) and Ag/AgCl electrode were used as counter and reference electrodes, respectively.

### 2.3. EIS Measurements

The electrochemical impedance spectrum (EIS) measurements were performed on PARSTAT 4000 (Princeton Applied Research, Oak Ridge, TN, USA) with conventional three electrodes system. Nickel disk electrode (diameter of 2 mm) and Ni(OH)_2_/Ni electrode (diameter of 2 mm) were used as working electrodes. Platinum foil electrode (1 cm × 1 cm) and Ag/AgCl electrode were used as counter and reference electrodes, respectively.

### 2.4. XPS Measurements

Elemental depth profiles were acquired by X-Ray photoelectron spectroscopy (XPS, ESCALab220i-XL, VG Scientific, East Grinstead, UK). The argon sputtering rate was 1 nm/min compared with Ta_2_O_5_ and the base pressure was about 3 × 10^−9^ mbar. The binding energies were referenced to the C 1s line at 284.6 eV from adventitious carbon. 

### 2.5. Detection of Sweet Substances

Cyclic voltammetry and chronoamperometry methods based on the same three electrodes system were used for detection and carried in the quiescent electrolyte solution of 0.1 M NaOH which contains 0.1 mM different kinds of sweet substances. The voltage ranged from 0.2 V to 0.6 V and scan rate was 50 mV/s.

The chronoamperometry method was also used to study the relationship of the characteristic value of this sensor and sweet substances’ concentration and relative sweetness.

### 2.6. Correlation with Human Sensory Sweetness

Pear, grape, orange, lemon, pitaya, and grapefruit were prepared to juices for the purpose of evaluating the sweet taste sensor’s correlation with human sensory sweetness. 200 g of above six kinds of fruits were washed clean, crushed five minutes by juicer with 200 mL water, and sieved, respectively.

Sensory evaluation score was measured by 10-member trained panel, based on the ranking method. The panelists were asked to evaluate the six samples and rank them according to their sweetness. After ranking, each sample in the order was assigned a score (1 means the weakest sweetness and 6 means the strongest). The sum score of each panelists was used as human sensory sweetness.

For comparison of the correlation, Brix and sweet taste sensor characteristic values of fruits were also measured by abbe refractometer and the newly built sweet taste sensor, respectively. 

### 2.7. Stability Evaluation of Sweet Taste Sensor

A 0.1 M NaOH solution and a 0.1 M NaOH solution containing with 2.4 mM glucose were used as the control solution and test sample, respectively, and were detected by sweet taste sensor every four days via cyclic voltammetry for 40 days. The signal ratio was calculated according to the following reaction:

(1) $$\mathrm{Signal}\;\mathrm{ratio}\;=\;\frac{I_{g\;}^{\prime }{/I}_{0\;}^{\prime }}{I_{g}{/I}_{0}}\;\times \;100\%$$

where $I_{g}$ was the initial day’s anodic peak current of test sample, $I_{0}$ was the initial day’s anodic peak current of control solution. The $I_{g\;}^{\prime }$ was the every four days’ anodic peak current of test sample, $I_{0\;}^{\prime }$ was the every four days’ anodic peak current of control solution. 

## 3. Results and Discussion

### 3.1. Fabrication of Sweet Taste Sensor

**Figure 1:**
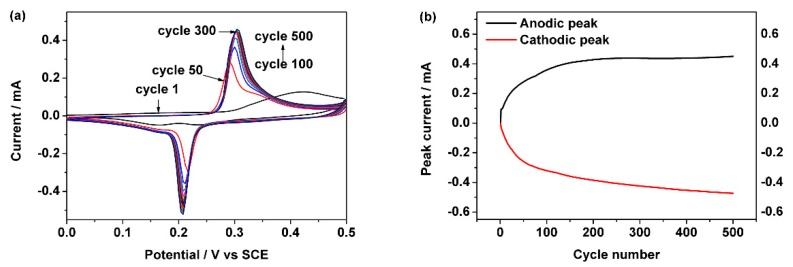
(**a**) Consecutive of cyclic voltammograms acquired at a scan rate of 100 mV/s for 500 cycles in 6 M NaOH solution. (**b**) Peak currents’ plots of anodic and cathodic in (**a**).

The new sweet taste sensor was fabricated by cyclic voltammetry technique. Figure 1a shows the results of consecutive cyclic voltammograms acquired at a scan rate of 100 mV/s for 500 cycles in 6 M NaOH solution. The stable cathodic peak and anodic peak are at 0.21 V and 0.31 V, respectively. The anodic peak current and cathodic peak current values of this sensor are similar to those of previous works [29,30,31]. Some small differences such as the stabilization cycle number may be caused by the surface area of nickel electrode, concentration of alkaline or acidic, and scan rate of cyclic voltammetry.

Figure 1b shows the cathodic peak current value and anodic peak current value of every segment in Figure 1a. The anodic peak current value rises at the first 300 cycles and reaches 430 μA. After 300 cycles, the anodic peak current value no longer increases and reaches a stable station, further cycles cannot increase current. 

During recent years, several studies have described the nickel (or with other metals) electrode’s electrochemical behavior in an alkaline solution [29,32,33,34,35]. The metal Ni changed to Ni(OH)_2_ in the alkaline solution according to the following reaction [36,37]:

Ni + 2OH^−^ → Ni(OH)_2_ + 2e^−^ (2)

Additionally, previous studies have demonstrated that the redox couple of Ni^2+^/Ni^3+^ was according to the following reaction [24,38]:

Ni(OH)_2_ + OH^−^ ⇄ NiOOH + H_2_O + e^−^ (3)

In Figure 1b, the anodic peak current and the cathodic peak current were almost the same, this phenomenon conformed to the Reactions (2) and (3). Thus, we think that the Ni(OH)_2_/Ni electrode has been fabricated. Therefore, a scan rate of 100 mV/s for 300 cycles in 6 M NaOH solution will be used as the standard process in following experiments.

**Figure 2:**
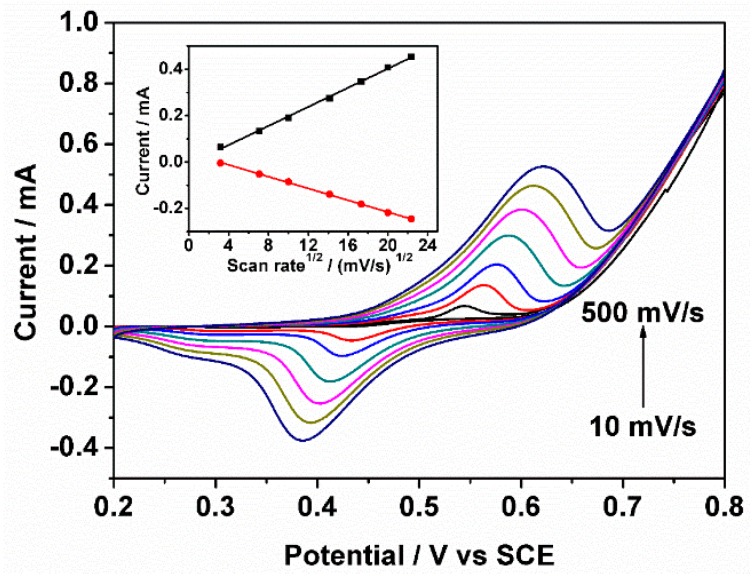
Cyclic voltammograms at different scan rate ranging from 10 to 500 mV/s in 0.1 M NaOH solution with addition of 2.5 mM glucose. The inset figure shows the linear dependence of the anodic and cathodic peak currents on the square root of the scan rate.

In addition, Figure 2 shows the relationship between the peak of response current and the scan rates, that is, 10, 50, 100, 200, 300, 400, and 500 mV/s in 0.1 M NaOH solution with 2.5 mM glucose. The redox couple exhibits an increased current response with the increase of scan rate from 10 mV/s to 500 mV/s. Furthermore, the anodic and cathodic peak currents exhibit an excellent linear relationship with the square root of the scan rates, indicating a typical diffusion-controlled electrochemical process [39,40].

### 3.2. Electrochemical Impedance Spectrum

**Figure 3:**
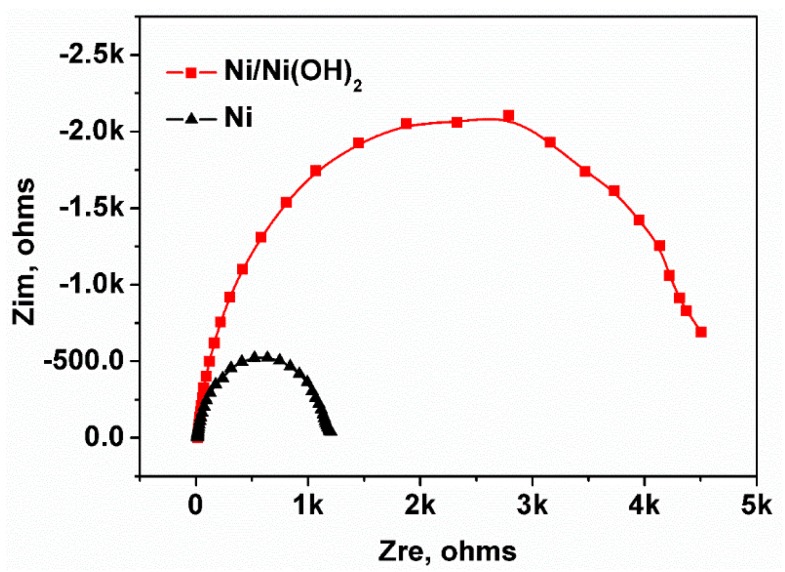
Nyquist impedance plots of Ni electrode and Ni(OH)_2_/Ni electrode.

The electrochemical impedance spectrum (EIS) measurement was used to detect the resistance of the electrode. EIS experiments were performed on PARSTAT 4000 in the 0.1 M KCl solution containing 0.05 M K_3_Fe(CN)_6_. The start frequency and end frequency were 100,000 Hz and 1 Hz, respectively. Amplitude was 10 mv RMS and was performed with open circuit. Figure 3 shows the Nyquist impedance plots of Ni electrode and Ni(OH)_2_/Ni electrode. As shown in the figure, Ni(OH)_2_/Ni electrode has wider loop size and higher polarization resistance than the untreated Ni electrode, indicating that the Ni(OH)_2_/Ni electrode has a larger electrochemical impedance after being fabricated from the Ni electrode [41,42]. 

### 3.3. Surface Characterization

**Figure 4:**
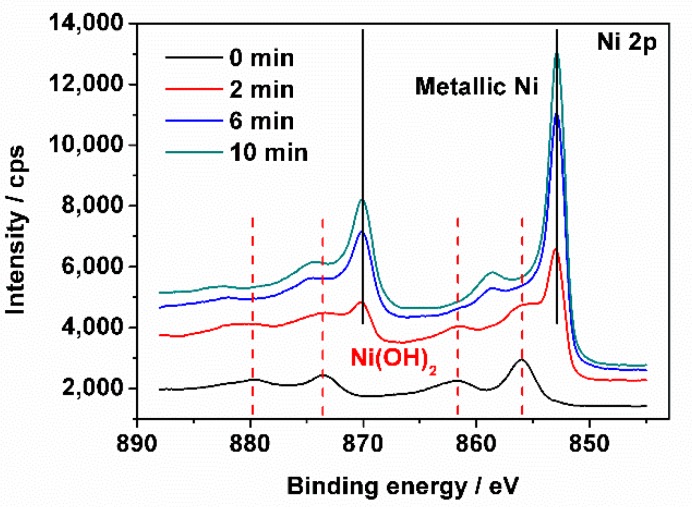
High-resolution X-Ray photoelectron spectroscopy (XPS) spectra of Ni 2p of the sweet taste sensor at different sputtering time.

X-Ray photoelectron spectroscopy (XPS) experiment was used to get more information on the sensor. Figure 4 shows the elemental depth profiles of Ni 2p spectra of the novel sweet taste sensor acquired by XPS at the different sputtering times. On the taste sensor surface (0 min), a peak at 856.2 eV indicates that Ni mainly exists in the form of Ni(OH)_2_. After sputtering for 2 min, peaks of metallic Ni at 852.9 and 870.2 eV begin to appear, and a peak at 856.2 eV also exists. This phenomenon indicates that Ni exists in two forms of Ni(OH)_2_ and metallic Ni at this depth. After sputtering for 6 min and 10 min, metallic Ni becomes dominant form of the taste sensor. Thus, the thickness of Ni(OH)_2_ layer fabricated by the chemical deposition is nearly 6 nm. 

### 3.4. Detection of Sweet Substances

Previous studies have demonstrated that NiOOH would transform into Ni(OH)_2_, while the alkaline solution contains some specific organic compounds according to the following reaction [24]:

NiOOH + organic compound → Ni(OH)_2_ + product (4)

**Figure 5:**
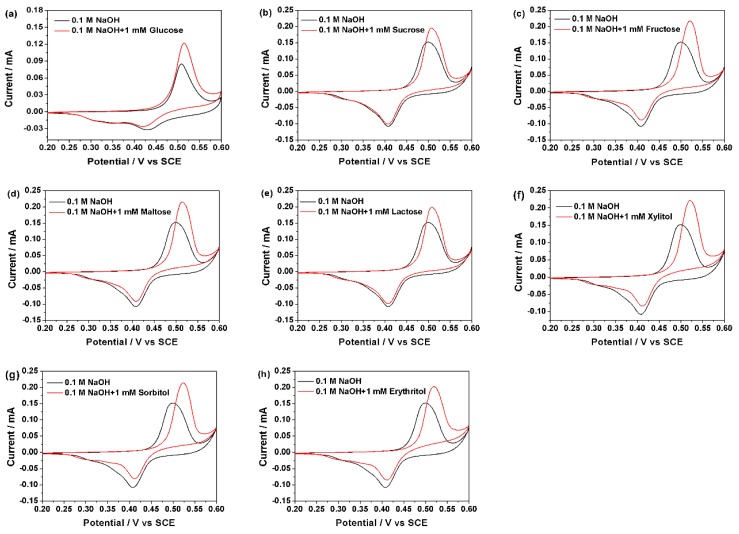
Cyclic voltammograms acquired at a scan rate of 50 mV/s in 0.1 M NaOH solution and 0.1 M NaOH solution with addition of 1 mM (**a**) Glucose; (**b**) Sucrose; (**c**) Fructose; (**d**) Maltose; (**e**) Lactose; (**f**) Xylitol; (**g**) Sorbitol; and (**h**) Erythritol.

Thus, the newly built sensor can be used to detect organic compounds, and in this study, the sensor was used to detect the sweet substances in NaOH solution. Figure 5 shows the obvious and good CV response of the sweet taste sensor in the 0.1 M NaOH solution containing eight kinds of different natural sweet substances. Compared with bare 0.1 M NaOH solution, the redox peak values of sweet substances were obviously raised and at the same time, the oxidation peak moved slightly toward the positive direction. According to Reactions (3) and (4), NiOOH, which was a result of the cyclic voltammograms, reacted with sweet substances and reduced to Ni(OH)_2_. Further, this increased concentration of Ni(OH)_2_, whose participation in the reaction would lead to increased oxidation peak value and delayed oxidation peak current potential. Thus, the sweet taste sensor exhibits a good response to the glucose, sucrose, fructose, maltose, lactose, xylitol, sorbitol, and erythritol. 

In addition, different natural sweet substances exhibited different anodic peak potentials and current values. At the concentration of 1 mM in 0.1 M NaOH solution, anodic peak potentials of glucose, sucrose, fructose, maltose, lactose, xylitol, sorbitol, and erythritol were 0.502 V, 0.507 V, 0.521 V, 0.514 V, 0.508 V, 0.519 V, 0.523 V, and 0.52 V, respectively. While the anodic peak current values were 0.1796 mA, 0.1870 mA, 0.2086 mA, 0.2090 mA, 0.1953 mA, 0.2047 mA, 0.2011 mA, and 0.2003 mA, respectively. Thus, the novel sweet taste sensor can respond to different kinds of natural sweet substances.

**Figure 6:**
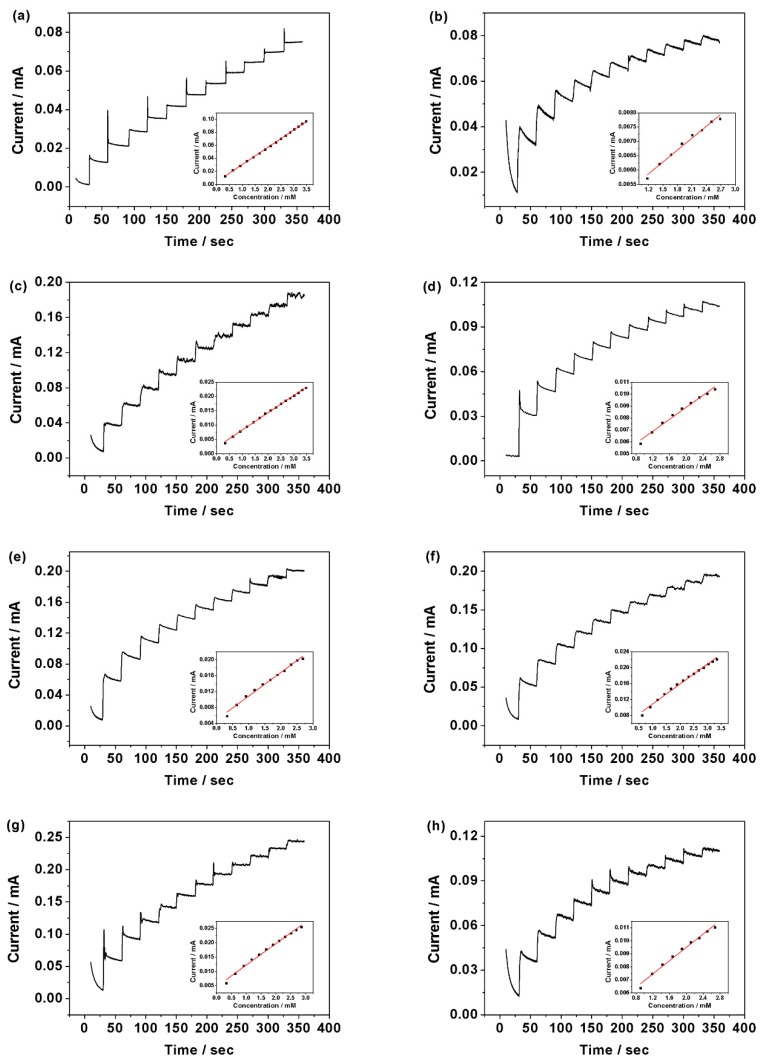
Amperometric response of the sweet taste sensor at 0.5 V versus SCE in 0.1 M NaOH solution with successive addition of 0.01 mM (**a**) glucose; (**b**) sucrose; (**c**) fructose; (**d**) maltose; (**e**) lactose; (**f**) xylitol; (**g**) sorbitol; and (**h**) erythritol.

The further response of sweet taste sensor to natural sweet substances was investigated by chronoamperometry. Two-hundred microliters of 0.05 M sweet substances was titrated into 30 mL of 0.1 M NaOH solution per 30 seconds with magnetic stirring and the applied potential was at 0.5 V. Figure 6 shows the amperometric response of sweet taste sensor towards to the glucose, sucrose, fructose, maltose, lactose, xylitol, sorbitol, and erythritol in 0.1 M NaOH solution, respectively. Fast and sensitive detection of these natural sweet substances was shown in the figure because of the instantaneous current responses and quick steady state—nearly shorter than one second. The corresponding calibration curves of Figure 6a–h exhibit a linear relationship between the eight natural sweet substances and the current value measured by the sweet taste sensor. The detection and performance parameters of glucose, sucrose, fructose, maltose, lactose, xylitol, sorbitol, and erythritol were listed in Table 1. The limit of detection (LOD) of the sweet taste sensor to glucose, sucrose, fructose, maltose, lactose, xylitol, sorbitol, and erythritol was 29.3 μM, 47.5 μM, 22.8 μM, 34.4 μM, 32.8 μM, 29.3 μM, 33.3 μM, and 33.7 μM, respectively. In the respective linear range, eight kinds of natural sweet substances all had good correlation coefficients (R^2^ > 0.98). 

In previous studies, in some sensors that used to detect glucose, sucrose, fructose, maltose, lactose, xylitol, sorbitol, and erythritol, the LOD and linear range were similar to this work [17,18,20,38,43,44,45,46,47,48]. In those research works, the detection emphasis of the sensor was on lower LOD, because these sensors were mainly used to detect glucose, lactose, or other substances in blood, serum, and interstitial fluid with the purpose of biochemical detection. However, in our research work, the detection emphasis of sensor has some differences with previous studies. This is because the sensor was used to detect natural sweet substances in food, such as juice, fruit, and soft beverages. Thus, the detection emphasis was broad-spectrum property of detecting several substances by single sensor at the same time. As Table 1 shows, the new built sweet taste sensor can widely respond to eight kinds of sweet substances and has good correlation coefficients.

In other studies, the researchers used the processed data for taste substances detection, such as reference value and CPA value [49,50,51]. The CPA value (V_r_’ − V_r_) was changed by membrane potential caused by absorption, which was based on the potential difference in the reference solution before and after absorbing the test sample. In this research, we established the characteristic value of the sweet sensor based on the current differences before and after adding the test sample, which could be calculated according to the following reaction:

(5) $$\;\mathrm{Characteristic}\;\mathrm{value}\;=\;I_{S}-I_{0}\;$$

where $I_{0}$ is the 120th second’s current value of 30 mL 1 M NaOH solution measured by the sweet taste sensor via chronoamperometry method, and $I_{s}$ is the 60th second’s current value of 30 mL 1 M NaOH solution containing the test sample.

**Figure 7:**
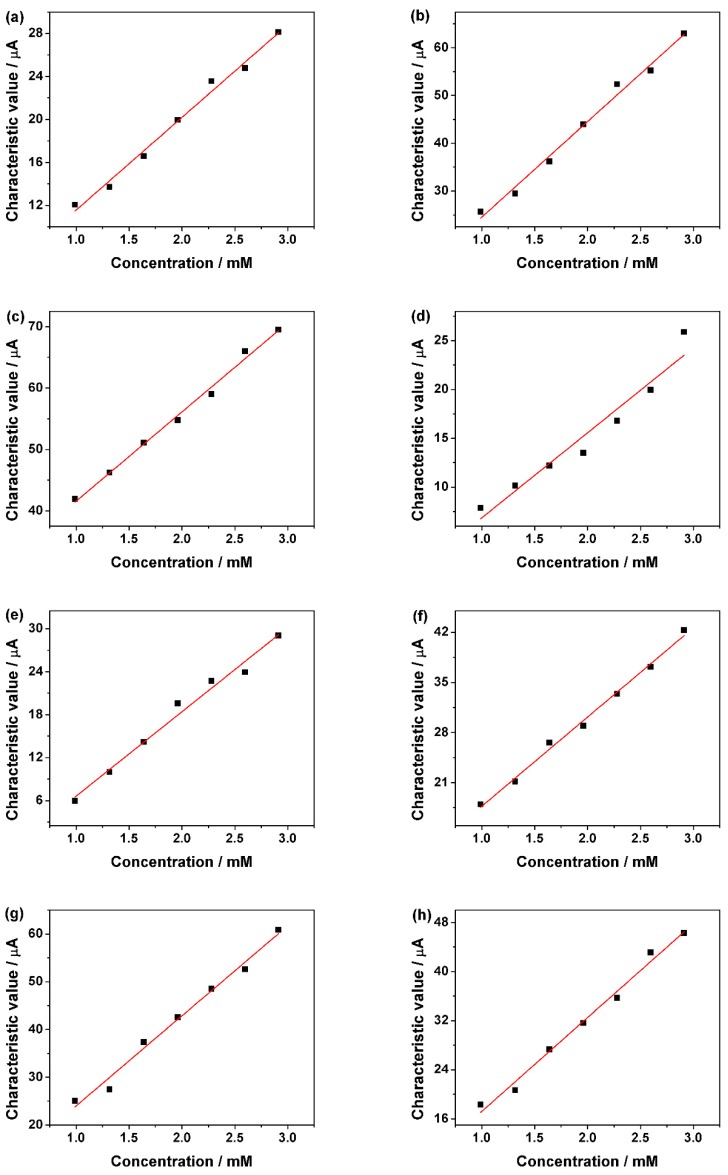
Correlation of the characteristic value measured by the sweet taste sensor in 1 M NaOH solution with difference concentration of (**a**) glucose; (**b**) sucrose; (**c**) fructose; (**d**) maltose; (**e**) lactose; (**f**) xylitol; (**g**) sorbitol; and (**h**) erythritol.

Figure 7a–h, the relationship of the characteristic value measured by sweet taste sensor and glucose, sucrose, fructose, maltose, lactose, xylitol, sorbitol and erythritol in 1 M NaOH solution, respectively, is shown. The fitted equations and correlation coefficients were listed in Table 2. All eight sweet substances exhibit a good linear relationship between the concentration and the characteristic value the sweet taste sensor. 

**Figure 8:**
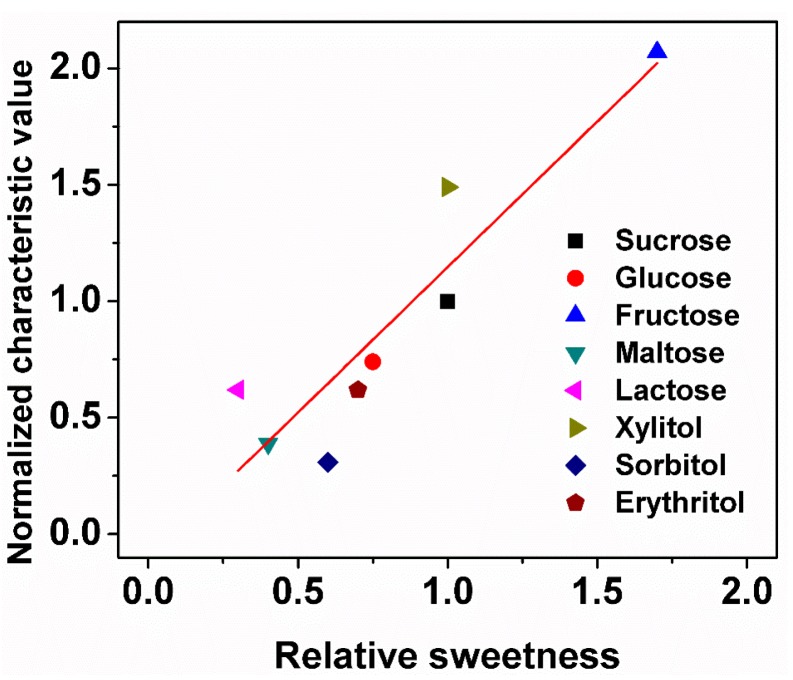
The correlation of characteristic value measured by the sweet taste sensor and relative sweetness of eight sweet substances.

Sweet substances all have their own sweet taste sensation intensity to human, which is named as sweetness [52,53]. Sweetness is a major contributor to the human palatability and also may influence the appetite [54]. It is also one of the most important aspects in some food [55,56]. In the food industry, sucrose is used as the reference sugar, and compared to sucrose, each sweet substance has its individual relative sweetness, which was measured by the human sensory evaluation. In order to study the relationship between relative sweetness and characteristic value, we use the 2mM of glucose, sucrose, fructose, maltose, lactose, xylitol, sorbitol, and erythritol in 1 M NaOH solution as test samples. Figure 8 shows the relationship of the characteristic value measured by sweet taste sensor and the relative sweetness of the eight sweet substances. On the X-axis is the relative sweetness of eight sweet substances based on sucrose’s sweetness and on the Y-axis is the normalized characteristic value of eight sweet substances based on sucrose’s characteristic value. The correlation coefficient between relative sweetness and normalized characteristic value was 0.81. The fact that it is closer to the fitted line implies a better match to human sensory sweetness. It is noted that the correlations of glucose, fructose, sucrose, maltose, and erythritol were good. Although for lactose, sorbitol, and xylitol, the correlations were not very good. The reason might be that the sensory sweetness of humans was not very equal to the taste senor and required data correction in a later study.

The above results indicate that the taste sensor and the characteristic value can be applied on the field of sweet taste detection. On the other hand, because of the broad-spectrum, in some other conditions, it may influence our sweet taste detection result at the complex samples, which contain many organic compounds, except for the natural sweet substances. Thus in the further study, we will focus on the selectivity of this sensor.

### 3.5. Correlation with Human Sensory Sweetness

For the purpose of researching the correlation with human sensory sweetness, the characteristic values of the sweet taste sensor from fruit juices were compared to human sensory evaluation. On the other hand, Brix of fruit juices were also measured by abbe refractometer at the same time. Table 3 lists the characteristic value, human sensory sweetness, and Brix of six kinds of fruit juices.

**Figure 9:**
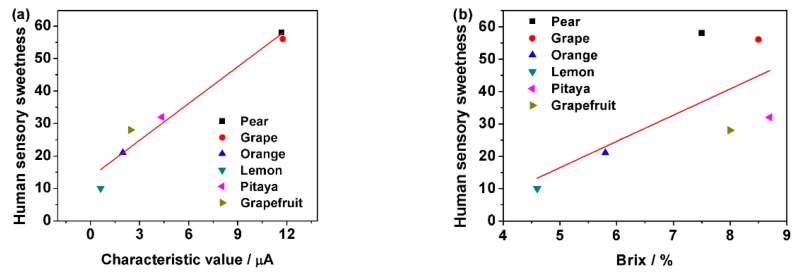
The correlation of human sensory sweetness score of six fruits and (**a**) characteristic value measured by sweet taste sensor; (**b**) Brix measured by abbe refractometer.

Figure 9a shows the correlation of human sensory sweetness of six fruit juices and characteristic value measured by the sweet taste sensor. On the other hand, Figure 9b shows the correlation of human sensory sweetness of six fruit juices and Brix. The correlation coefficients of Figure 9a,b were 0.95 and 0.35, respectively. The correlation coefficients indicate that the sweet taste sensor’s characteristic value has good correlation with human sensory sweetness and Brix’s correlation was not very good in this experiment.

Nowadays, Brix is widely used in food industry because of quick and conveniently reflection of the concentration of dissolved sweet substances in liquid food. However, for complex liquid, Brix would not only reflect the concentration of sweet stances, but also reflect other dissolved substances such as starch, cellulose, and protein. Thus, Brix could not truly reflect the sweetness of samples. Compared with Brix, the characteristic value measured by the new built sweet taste sensor, which was broad-spectrum, responded to mostly natural sweet substances in liquid food samples and was not affect by other dissolved substances. Thus, it could well and truly reflect the sweetness of samples evaluated by human sensory.

### 3.6. Stability of Sweet Taste Sensor

**Figure 10:**
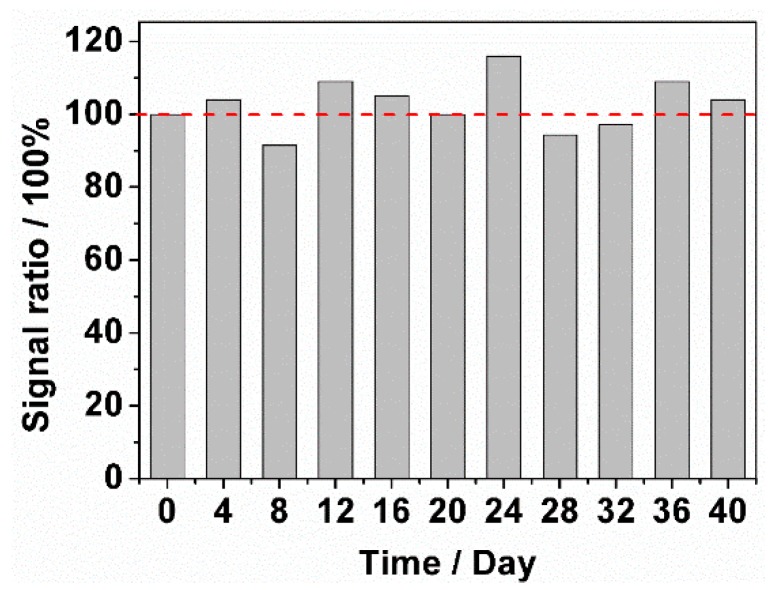
Stability of the sweet taste sensor stored under dry and ambient condition towards 0.1 M NaOH solution containing with 2.4 mM glucose monitored every four days for 40 days.

Stability is also one of critical requirements for sweet taste sensor. The novel sweet taste sensor was used to detect the current response of pure 0.1 M NaOH solution and 0.1 M NaOH solution containing 2.4 mM of glucose every four days for 40 days via cyclic voltammetry. Further, when not in use, this sensor should be sealed with a cap. Figure 10 shows the stability result of 40 days with the interval of 4 days. During the 40 days, the sensor’s signal ratio exhibited a small fluctuation, ranged from 91.5% to 116.2%. Thus, the sensor has a well stability of detection during the long-term. On the other hand, when the signal ratio is less than 70%, the signal of test solution (0.1 M NaOH solution + 2.4 mM glucose) was closed to the control solution’s signal (0.1 M NaOH). Thus, the sensor was not expected to detect. Based on this, we think the sensor should be refabricated in the 6 M NaOH solution at this condition. The signal ratio of sensor decreased to 63.4% at the 48th day. Therefore, we establish that this sensor should be condition in 6 M NaOH solution by application of CV technique at a frequency of 48 days.

## 4. Conclusions

A broad-spectrum sweet taste sensor based on Ni(OH)_2_/Ni was fabricated by cyclic voltammetry technique in the NaOH solution. This sweet taste sensor exhibits well broad-spectrum and linear range response to glucose, sucrose, fructose, maltose, lactose, xylitol, sorbitol, and erythritol (R^2^ = 0.998, 0.983, 0.999, 0.989, 0.985, 0.990, 0.991, and 0.985, respectively). Based on this, we established the characteristic values of the sweet sensor, which have good correlation with the sweet substances’ concentration and relative sweetness. Compared with Brix measured from abbe refractometer, characteristic values measured from the sweet taste sensor has better correlation with human sensory sweetness (correlation coefficient = 0.95), and can better reflect nature sweetness of samples. In addition, the sweet taste sensor also exhibits a well long-term stability during 40 days. Thus, this broad-spectrum taste sensor will be promising for sweet taste sensory application. 

## Figures and Tables

**Table 1:** Detection and performance parameters of sweet taste sensor to eight sweet substances in 0.1 M NaOH solution by chronoamperometry. LOD—limit of detection.

Natural Sweet Substances	LOD (μM)	Linear range (mM)	R^2^
Glucose	29.3	0.323–3.478	0.998
Sucrose	47.5	1.176–2.683	0.983
Fructose	22.8	0.323–3.478	0.999
Maltose	34.4	0.323–2.683	0.989
Lactose	32.8	0.909–2.683	0.985
Xylitol	29.3	0.625–3.333	0.990
Sorbitol	33.3	0.323–2.857	0.991
Erythritol	33.7	0.909–2.683	0.985

**Table 2:** The fitted equations and correlation coefficients of sweet taste sensor to eight sweet substances.

Natural Sweet Substances	Fitted Equation *	R^2^
Glucose	y = 8.62x + 2.96	0.988
Sucrose	y =20.01x + 4.54	0.988
Fructose	y =14.53x + 27.06	0.993
Maltose	y = 8.71x − 1.85	0.936
Lactose	y = 11.78x − 5.14	0.981
Xylitol	y = 12.45x + 5.26	0.993
Sorbitol	y = 18.84x + 5.19	0.985
Erythritol	y = 15.28x + 1.94	0.988

* x (mM) is the concentration of sweet substance and y (μA) is the characteristic value measured by the sweet taste sensor.

**Table 3:** Characteristic value, human sensory sweetness, and Brix of six kinds of fruit juices.

Fruit Juices	Characteristic Value (μA)	Human Sensory Sweetness	Brix (%)
Pear	11.69	58	7.5
Grape	11.76	56	8.5
Orange	1.98	21	5.8
Lemon	0.63	10	4.6
Pitaya	4.37	32	8.7
Grapefruit	2.49	28	8.0
